# Supplementary material for: Are carbon emissions trading and green financial instruments synergistic? -Comprehensive quantitative research based on content analysis
Source: PLoS One. 2024 Mar 7;19(3):e0298601. doi: 10.1371/journal.pone.0298601 (PMC10919601; doi:10.1371/journal.pone.0298601)
Supplement: S1 Data — (ZIP) [file pone.0298601.s001.zip › Data/地级市Z_F工作报告.zip!/╟α║ú/╬≈─■╩╨/└┤╫╘╣1⁄2╓┌║┼í╛┬φ┐╦╩2╛▌═°í┐_2012.docx]

各位代表：

现在，我代表市人民政府向大会作工作报告，请予审议，并请市政协各位委员和列席会议的同志提出意见。

一、2011年工作回顾

2011年，在省委、省政府和市委的坚强领导下，全市各族人民以科学发展为主题，以加快转变经济发展方式为主线，以"四个发展"为主要路径，抢抓机遇，开拓创新，攻坚克难，锐意进取，全面完成了市十四届人大七次会议和市十五届人大一次会议确定的各项任务，奠定了"十二五"发展的坚实基础。

----经济发展持续向好。全年实现地区生产总值770.7亿元，增长15%；地方一般预算收入45.3亿元，增长31.1%；完成地区固定资产投资528亿元，增长31 %；社会消费品零售总额271.3亿元，增长17.1%。发展方式不断转变，综合实力再上新台阶。

----产业结构不断优化。三次产业比重调整为3.6∶53.4∶43.1，其中一产比重较上年降低0.3个百分点，二产比重提高2.3个百分点，对经济增长的贡献率达到62.7%，工业主导型产业结构得到进一步强化，园区引领作用尤为明显。服务业规模迅速扩大，旅游及现代服务业快速发展。

----社会民生显著改善。实现城镇居民人均可支配收入15842元，增长12.5%；农民人均纯收入6634元，增长21.4%，人民生活水平进一步提高。积极采取临时价格干预和财政补贴等多种措施，有效抑制物价高位运行，切实保障了困难群众的生活。全年用于社会民生的投入占到财力总支出的80%以上，城乡群众的生产生活条件大为改善，人民群众的幸福感不断增强。

----城乡面貌日新月异。统筹新区建设和旧城改造，加大基础设施建设力度，完成投资55亿元，一批关乎长远、涉及民生的重点工程相继实施和建成，城乡面貌发生了显著变化。

在具体工作中，我们重点抓了以下八个方面：

（一）强化项目拉动作用，投资结构逐步优化。围绕东部城市群建设的目标要求，加大固定资产投资力度，实施一批重大项目，巩固和保持了经济持续增长的良好势头。严格实行重点建设项目市级领导联系督导制度，加强对火车站综合改造、保障性住房建设、教育布局调整、排污口整治等23项重点项目的督查，确保了项目顺利实施。不断扩大投资规模，优化投资结构，涉及民生项目的投资大幅增长，其中医疗卫生、节能环保、农林水务等方面的投资分别增长51.4%、40.3%和39.6%。进一步拓宽投融资渠道，民间投资日益活跃，投资比重达到65.2%。加大招商引资工作力度，到位资金176亿元，其中省外到位资金119亿元。"青洽会"签订各类项目33个，签约金额189.5亿元，外向型发展水平进一步提高，全方位开放的成果日益丰硕。

（二）突出特色加快发展，结构调整深入推进。我们始终把调整优化产业结构作为加快转变经济发展方式的必然路径，大力实施"工业强市"战略，投资242.9亿元，实施工业项目177项，工业技术创新项目32项。多晶硅、单晶硅、电子铝箔、镁合金、藏毯绒纺、生物制药、改装汽车等一批重点工业项目建成投产，铝板带箔、PVC复合材料、二甲醚、磷酸铁锂正极材料、光伏电池及组件等一批重大项目建设进展顺利，对优化产业结构、提升产业层次、打造优势产业集群、增强发展后劲发挥了重要作用。全力支持工业园区做大做强，开发区完成工业增加值197.8亿元，获得省政府"2011年度青海省工业投资贡献奖"，对全市工业经济拉动作用进一步增强。大力推进以旅游业为龙头的第三产业，加快旅游基础设施建设和特色旅游产品开发，组团赴韩国、澳大利亚等开展了大型推介活动，全年共接待国内外游客981.3万人次，实现旅游总收入56.2亿元，西宁被世界科学发展联盟和联合国国际减灾战略署评为"世界旅游名城"。以现代物流、金融保险、信息服务等为主的现代服务业快速发展，朝阳物流园区、铁路物流中心基本建成，物流业基础进一步夯实。

（三）扎实推进"三农"工作，城乡统筹成效明显。全面落实强农惠农措施，大力发展现代设施农业，农业生产喜获丰收，粮食总产量26.9万吨，蔬菜总产量72万吨；肉、奶、禽蛋产量分别增长6.4%、10.8%、20.9%。集中建设冬暖式温室3500栋，建设畜棚4000栋，新（改、扩）建各类养殖基地75个。加快推进农业产业化，市级以上龙头企业达到153家，带动周边农户21万户。认真实施"千村建设"、"百村示范"计划，农村水利、公路等一批基础项目顺利实施；完成村道硬化360公里，新建农村公路49公里；72个贫困村整村推进项目全面完成，实现4.7万人稳定脱贫。城乡统筹工作力度加大，"三农"工作取得显著成效！

（四）不断完善城市功能，承载能力逐年增强。扎实推进"东延、西扩、南活、北优、中疏"的发展战略，全面完成了《西宁市2030年城市空间总体发展规划》及各类专项规划和城市重点部位、重点片区、重点项目的详规修编工作。旧城改造稳步进行，完成拆迁面积106.4万平方米，开发建设1399万平方米。规范房地产市场秩序，稳步推进房地产开发建设，商品房施工面积1276万平方米。加大供排水设施建设，新增管网27.3公里，改造管网11公里，供排水能力不断提高。深入实施畅通交通工程，人行过街天桥、地下通道、停车场等项目进展顺利，道路通行率平均提高20%，有效保障了安全有序顺畅出行。推进重点功能区的开发建设，海湖新区完成固定资产投资62亿元，生态宜居形象初步显现。做强西宁，服务全省的目标正在大步推进！

（五）积极实践绿色发展，生态环境进一步改善。强化生态环境建设，创卫、创绿成果得到有效巩固。重点绿化工程按计划实施，完成大南山生态绿色屏障造林1.7万亩，各项工程造林27.3万亩，义务植树501.2万株，新增园林绿地40公顷，完成海棠公园、河湟公园等主题公园建设。围绕国家环保模范城市创建，启动了"清水入城"工程，开展湟水河排污口综合整治和南川河城区主河道清淤，停产、取缔了一批违规采砂场。历史遗留铬渣处置、"两废"中心建设等一批环保重点项目进展顺利。6个示范片区、83个村的农村环境连片综合整治项目有效实施。推进循环经济和清洁生产，淘汰了一批落后产能，严格控制污染物排放，全年空气质量优良天数316天，比上年增加5天；湟水河小峡出口断面水质达到三类，全面完成了省政府下达的节能减排目标，城市宜居环境得到进一步改善！

（六）各项改革纵深推进，体制机制更加有效完善。全面落实深化医药卫生体制改革的决策部署，实行政事分开、医药分开，成立了市医疗卫生服务管理中心、市医疗机构药事服务监管中心，医药卫生体制改革取得阶段性成效。大力培育市场主体，组建了湟水投资管理公司、园林旅游体育资产经营公司和公共租赁住房发展运营管理公司，市场化运营体系不断完善。深化投融资体制改革，农村商业银行正式挂牌营业，浦发银行、新华寿险等一批金融机构在西宁落户。继续推进城投公司二期企业债券发行工作,融资能力明显增强。深化行政体制改革和机关人事制度改革，制定并试行了市政府常务会议市民旁听、涉及民生的听证会现场直播、政府雇员、总师等制度。加快市级公园管理改革，完善管理制度，成立了公园管理中心，对5个市级公园全面实行了免费开放。深化文化体制改革，文化事业大发展、大繁荣的活力进一步激发，广大人民群众享受了更加丰富的精神文化生活！

（七）倾心关注民生福祉，群众幸福指数稳步提升。坚持把新增财力的80%和主要工作精力放在社会民生事业上，把就业作为民生之本，不断激发全社会创业、就业的激情与活力，城镇新增就业2.7万人，各类失业人员就业1.9万人，农村劳动力流动就业35.1万人次。社会保障覆盖面持续扩大，养老保险新增扩面13616人，失业保险扩面4300人；新型农村养老保险参保率达到92%，城镇居民养老保险顺利启动，实现了社会保障制度的全覆盖；开工建设各类城镇保障性住房31905套，农村奖励性住房30250户，农村危旧房改造18000户。大力推进教育布局调整和校安工程建设，改造学校危房33万平方米，湟川中学、西宁二十一中新校区交付使用；"两基"工作顺利通过国家验收，教育布局调整、素质教育等多项成果走在了全国前列。加快医疗卫生基础设施建设，市中医院门诊综合楼等建成使用，城乡医疗卫生设施条件进一步改善。积极推进农村基层文化为民办实事工程，建成352个农家书屋和11个乡镇文化站。扎实推进全国文明城市创建工作，获得"2011年全国文明城市提名资格"，我市有1个村、11个单位分别获得"全国文明村镇"和"全国文明单位"荣誉称号。稳步推进国家创新型城市建设，落实"5568"创新工程各项任务，全面启动农业科技示范园区建设，获得"全国科技进步先进城市"殊荣。

市委、市政府10大类62项为民办实事项目顺利完成，全面兑现了向全市人民的庄严承诺！

（八）加强民主法制建设，和谐稳定局面进一步巩固。切实加强依法行政，自觉接受人大法律监督和政协民主监督，一年来共办理人大代表建议158件，政协委员提案160件，办结率100%。全面落实政府系统党风廉政建设责任制，严格执行勤政廉政各项规定，廉政建设取得良好实效。进一步加强安全生产工作，安全生产形势持续稳定好转。强化食品药品监管和农产品及农药监管，开展滥用食品添加剂、饮食安全等专项整治工作，人民生命财产安全得到有效保障。扎实推进社会管理创新综合试点工作，积极探索建立与西宁经济社会发展相适应的社会管理组织体系和工作网络，公共管理服务水平进一步提升。认真开展"双拥"共建活动，人民解放军、武警官兵、公安干警在抢险救灾、维护社会稳定中发挥了重要作用。大力推行信访责任追究制度，有效化解了各类不稳定因素。认真贯彻党的民族宗教政策，各民族团结互助、共谋发展的局面得到巩固。不断加强工商、统计、气象、质监等工作，人口与计划生育、妇女儿童、残疾人等事业取得新的进步。

与此同时，我们坚决贯彻省委、省政府建设新玉树的重大部署，扎实做好援建工作，涉及杂多县萨呼腾镇经济发展、社会民生的62个援建项目进展顺利，完成投资7.2亿元，完成援建任务的66.1%，其中4个村的农牧民住房和城镇居民住房等8个项目已竣工并交付使用，得到了杂多县干部群众的肯定和好评。

各位代表，回顾一年来的政府工作，成绩来之不易。这些成绩的取得，是省委、省政府和市委坚强领导的结果，是全市各族人民艰苦奋斗与开拓进取的结果，是市人大及其常委会依法有效监督和市政协民主监督、鼎力支持的结果，在此，我代表市人民政府，向各位人大代表、政协委员，向各民主党派、工商联、无党派人士和各人民团体，向奋斗在全市各条战线、各行各业的建设者和劳动者，向中央驻宁单位、省直各部门、人民解放军驻宁部队指战员、武警官兵和公安干警，向所有关心、支持西宁建设和发展的各界人士，致以崇高的敬意和衷心的感谢！

在肯定成绩的同时，我们也清醒地看到，在全市加快发展的进程中还存在一些困难和问题，主要是：随着社会转型的加快和经济发展的加速，人口、资源、环境等方面的压力逐年增大；经济总量小、产业层次低、结构不合理的问题依然突出，财政规模小、调控能力弱的制约因素依旧存在；拆迁和安置的难度加大，制约了城市建设的快速发展；城市综合环境、功能品位与市民的期望相比还有一定差距；社会建设和文化建设还面临许多新课题，政府行政效能和服务水平与广大人民群众的要求还有一定差距，这都需要我们在今后的工作中努力加以解决。

二、2012年总体目标和主要任务

今年是"十二五"承上启下的关键一年。政府工作的指导思想是：全面贯彻党的十七届六中全会、中央经济工作会议和省委十一届十一次全体会议、市第十三次党代会和市委十三届二次全体会议精神，牢牢把握发展"主题"、"主线"和"主要路径"，坚持稳中求进、好中求快的方针，大力实施"一统三基"发展战略，围绕让人民生活更加幸福的目标，围绕"五个三"工程，加快优化需求结构、产业结构和区域结构，注重投资拉动和产业支撑，牢牢抓住城市规划建设、产业发展、文化提升、民生保障等重点，抓细节、做精品，全面加快"四个发展"，全力推进文明城市创建，用新举措丰富生活之城、幸福之城的新内涵。

经济社会发展的主要预期目标是：地区生产总值增长14%；地方一般预算收入增长17%；全社会固定资产投资增长20%；社会消费品零售总额增长16%；旅游总收入增长20%；城镇居民人均可支配收入增长10%，农民人均纯收入增长13%；居民消费价格涨幅控制在5%左右；城镇登记失业率控制在4.2%以内；农村转移劳动力30万人次；节能减排控制在省上下达目标以内。

各位代表，全面完成今年的目标任务，需要我们进一步解放思想，创新理念，改进工作方法。我们将牢固树立精细理念，悉心打造精品城市。不论是发展经济、还是改善民生，不论是城市建设、还是社会管理，都要体现精品意识、精细理念，从大处着眼、小处着手，盘活存量，提升效益，优化服务。坚持把心思聚在干事业上，把精力放在办实事上，把功夫下在抓落实上，把本领用在出精品上，精细化规划、精细化建设、精细化管理，精细化服务，让未来在规划中更加靓丽，让城市在建设中更具特色，让社会在管理中更趋和谐，让人民在服务中更加满意！

在今年的工作中，我们将重点突出以下八个方面：

（一）致力于增强经济发展后劲，突出实施重点项目。以"项目推进年"为契机，组织实施一批重大基础设施、商品房开发、生态环境保护、重点社会民生等领域592个建设项目，实现全社会固定资产投资增长20%，完成投资630亿元以上的目标。

准确把握投资重点。深入研判宏观经济形势，把握国家投资重点，有针对性地在水利、节能减排、保障性住房、扶贫开发、生态环境和改善民生等领域预先谋划、精心组织，及时申报一批项目，力争更多的项目列入国家和省上的计划，使项目成为经济社会发展的重要支撑。

继续优化投资结构。充分发挥政府投资引导作用，注重对改善"三农"的投入、对教育和公共卫生等社会事业发展的投入、对城乡居民住房和改善人居环境的投入、对流通领域基础设施的投入，鼓励和引导民间投资进入基础产业、基础设施、社会事业和现代服务业等领域。进一步强化招商引资的地位和作用，围绕产业定位和比较优势，综合运用土地、财税、金融等手段，积极引进战略投资者、新兴产业、知名品牌和重大项目，做好相关配套服务，确保招商引资重大项目落地实施。

加强项目实施监管。全面落实重点建设项目市级领导联系督导制度、重大项目工作例会制，严格项目监管，统筹安排，责任到人，争取早安排、早协调、早征地、早开工。保证建设有序，投资到位，质量过硬，力争使每个工程都成为效益显著、人民满意的工程。

同时，全力抓好玉树援建工作，圆满完成决战年的建设任务，为建设新玉树贡献应有的力量。

（二）致力于转变经济发展方式，不断调整优化结构。深刻认识和全面把握省委关于西宁要实现"三个新突破"的要求，把转变经济发展方式作为实现经济稳步发展的主攻方向，努力优化产业结构，构建具有西宁特点的现代产业体系，进一步激发产业发展活力，拓展发展空间，扩大经济总量，提高发展质量，为全面建设小康社会提供坚实的发展基础。

突出工业支撑作用。坚持"工业强市"战略，加快传统产业改造升级，大力发展新兴产业，做大做强特色产业，确保工业投资完成290亿元以上，工业增加值增长17%以上。抓紧制定落实国务院关于进一步支持小型和微型企业健康发展意见的具体措施，认真贯彻省政府八大产业调整和振兴实施意见及关于应对当前工业经济突出问题的十项举措，扎实推进"双百"行动，以引进大型企业和培育知名品牌为突破口，加快推进自主创新，集中力量培育和发展太阳能光伏制造、有色金属、特色化工、藏毯绒纺、高原生物制品、装备制造、锂资源精深加工等特色产业，进一步加快项目建设，延伸产业链，扩大产业规模，优化产业结构。积极为园区经济发展提供更优质的服务，使园区成为推动全市经济发展的强大支撑。充分利用县域工业园和资源禀赋条件，围绕产业定位和发展优势，因地制宜，加快发展，使其成为新的经济增长点。

提升服务业发展水平。扎实推进国家服务业改革试点城市建设，加大对旅游地产、旅游重点景区和重点项目、文化体育旅游产业的扶持力度，开发建设新型旅游产品，完善配套服务设施，全面加快旅游业发展。积极引进具有先进管理经验和优质高效服务的国际、国内知名品牌服务企业，大力发展连锁酒店、家庭旅店等服务业，提高旅游接待能力和服务水平。围绕海湖新区商业核心圈建设，启动夏都会议中心、北京新华联等项目建设，不断完善会展业基础设施和配套设施功能，建成青海国际会展中心C馆。做好商业巷、水井巷商务区、建国路等重点商圈建设，实施王府井城西店等商贸项目，提升商业中心档次和竞争力。继续加大投入力度，加快农副产品批发市场和专业市场、连锁便利店、社区菜店及配送中心改造等商贸流通设施建设，扶持和培育一批农副产品流通领域的龙头企业，抓好青藏高原农副产品集散中心的启动运营，及早发挥集散中心调控物价、保障供给的基础作用。

持续发挥消费拉动作用。积极寻找扩大投资与消费的结合点，以改善投资结构拓展消费空间，促进投资与消费良性互动，实现社会消费品零售总额315亿元。积极改善民生，促进居民创业就业，千方百计增加群众收入，提高养老、医疗、低保、住房等社会保障水平，努力增强消费预期。加强对文化体育旅游等消费热点的引导，不断培育新的消费增长点。巩固扩大"万村千乡"和"双百市场"建设成果，落实好家电下乡"提标扩面"等政策，努力扩大农村消费。多措并举，全力促进消费总量的不断扩大和消费水平的显著提升。

（三）致力于统筹城乡发展，持续促进农民增收。坚持不懈地推进城乡一体化建设，不断加快农业发展、农村繁荣、农民增收的步伐，逐步实现城市资源向农村延伸，公共服务向农村覆盖，城市文明向农村传播，促进城乡共同繁荣。

发展特色现代农业。按照率先在全省建成现代农业示范中心的目标，以推进农业科技创新，持续增强农产品保障供给能力为突破口，全面加快农业产业园区建设，推进都市休闲观光农业发展步伐。加强蔬菜、畜产品生产，提高农畜产品的商品率和自给率，特色种植比例保持在75%以上。加快新型设施农业建设步伐，新建冬暖式日光节能温室和大棚各3000栋，牲畜暖棚4000栋，新（扩）建奶源、肉牛（羊）、生猪基地各10个，禽类基地5个。新（扩）建蔬菜保鲜库5座，使全市的冷藏保鲜库容达到10万吨，提高菜篮子产品储存调剂能力。

夯实农业生产基础。加快以水利为重点的农业农村基础设施建设，做好"引大济湟"西干渠建设工程，进一步完善城区及县域供排水配套管网，抓好重点河段防洪和病险水库除险加固工程，实现水资源的可持续利用。着力抓好人畜安全饮水工程、设施农业和养殖基地水利设施建设，继续实施灌区改造工程，大力推广农业节水抗旱技术。统筹建设一批农村水、电、路、沼气、太阳能灶等项目，解决5万人、4.5万头（只）牲畜的饮水安全问题，建成农户沼气池6600座，新建农村公路100公里，硬化村道100公里，不断改善农村生产生活条件。

加快新农村建设步伐。加快推进"三集中"，力争完成土地集中经营25万亩，不断提高土地的利用率和产出率。加强组织领导，全面整合农村奖励性住房建设、易地扶贫搬迁、党政军企共建示范村等涉农项目资金，集中实施100个示范村建设,切实改善农村面貌。准确把握新阶段扶贫开发的大趋势，坚持政府主导、社会帮扶与农民主体作用相结合，普惠性与特惠性政策相配套，扶贫开发与社会保障相衔接，努力走出一条具有西宁特点的扶贫开发道路。今年集中实施60个贫困村整村推进项目，解决5万名贫困人口的温饱问题。发展壮大城郊经济、县域经济，着力打破市内四区三县之间资源和要素流动的各种障碍，推动区域内经济形成城乡结合、优势互补、一体化竞相发展的生动局面，促进城乡群众共同发展、共同繁荣。

（四）致力于增强载体服务功能，科学规划建设城市。贯彻东部城市群发展战略，努力增强西宁作为青藏高原现代化中心城市的功能。突出体现"现代、大气、特色"的城市品质，用规划控制和引导城市建设，构建适度超前、功能配套、安全高效的城市发展框架，逐步增强宜居、宜业、宜游、宜人的城市功能。

强化西宁引领作用。围绕转变发展思路、城市职能、发展方式，进一步突出西宁在东部城市群建设中承担的"核心引领"、"辐射带动"两大区域作用和"区域服务"、"区域组织"、"区域管理"三大区域职能，通过区域职能的合理分工、重大设施的协调布局和城市群网络发展的统筹等方法，构建区域一体化发展的新格局。

细化城市各层次规划。以《2030年城市总体规划》修编工作为契机，制定《西宁市城市规划管理技术规定》、《西宁市居住公共服务设施规划配套标准》等一系列地方规章与标准，落实城市各类公共服务设施的发展用地和空间，实现公共服务设施的"均等化"发展，全面提升城市规划建设水平和生活环境质量。加快重要街区修建性详细规划编制工作，努力将规划与城市发展进程、片区改造、城中村改造紧密结合起来，推动规划工作向精细化迈进。立足高原山水河谷城市特质，注重挖掘历史文化底蕴，加快第五立面研究和城市色彩导则制定，强化城市空间与自然山水格局的呼应，提炼和塑造西宁城市独有的特色。

进一步完善载体功能。继续推进"东延、西扩、南活、北优、中疏"发展战略，力争启动多巴综合片区建设。加强土地管理，合理利用有限的土地资源，进一步规范土地招拍挂和集体用地转为建设用地工作。抓好火车站综合改造、中心广场北扩等重点项目，打造精品街区。完善海湖新区服务功能建设，充分利用环湖赛、西宁青年电影节等重大活动推介海湖新区，尽快聚集人气。加快京藏高速南过境公路建设，启动凤凰山快速路项目，做好兰新铁路第二双线等重点工程的协调服务，提高西宁与周边重大交通枢纽的沟通衔接。抓好柴达木路综合整治二期等道路建设，持续抓好畅通交通工程，实施停车场、人行天桥、换乘中心、微循环片区、畅通示范街等项目，优化静态交通，分阶段实施环城公交、直达公交、同城公交建设，在有条件的道路设立公交专线，优化线路，提升服务水平，进一步提高畅通能力。做好城市排水设施维护工作，加大老城区老化管网改造力度，新建市区扩展和污水处理厂配套管网，全面保证城市"输血"功能的健康有序成长。

推进城市精细化管理。不断改进和完善工作，加强规范化、制度化长效管理机制建设，健全完善法规体系和工作机制，加强城市各个方面、各个环节的精细管理，持续巩固和发展创卫成果，确保顺利通过国家复检。扎实推进城乡环卫一体化，加快数字化城市管理信息系统建设。通过城市管理的科学化、精细化和人性化，让西宁更具亲和力！让人民更有幸福感！

（五）致力于提高可持续发展能力，不断改善生态环境。以创建全国森林城市和国家环保模范城市为目标，加快生态环境建设和保护，促进生活环境、建设环境以及投资环境的改善，努力建设高原山水花园城市。

大力推进生态建设。坚持"三环"绿化战略，抓好大南山生态绿色屏障、小酉山绿化工程，完成"三北"防护林、公益林等造林工程26.3万亩。加快城市出口道路绿化美化、城区"三河六岸"绿色滨河景观带、北山危岩体综合治理等城市重点景区及主题公园建设，抓好水土保持工程、水资源保护工程等项目。深入实施 "清水入城"工程，推进湟水流域水污染综合治理，加快再生水利用项目建设，开工建设第四和第五污水处理厂，力争湟水河出口断面水质逐年改善。

切实强化节能减排。进一步健全节能减排目标责任考核机制，支持和引导企业加快技术研发和技术改造，积极培育以低碳产业为特征的绿色发展模式，不断完善"有色金属——天然气——盐化工"产业链，构筑循环经济体系，提高清洁能源消费比重。倡导低碳绿色生活方式和消费模式，加强建筑节能监督和管理，推进可再生能源建筑应用工作，形成低能耗、低排放、低污染的生产生活新形态，推进低碳型社会建设。

扎实推进创模工作。不折不扣地落实政府、企业、个人的主体责任，全力完成"创模"达标各项阶段任务。实施以历史遗留铬渣综合整治为重点的重金属污染和工业废水处置工程，加快"两废"中心、餐厨垃圾综合利用等项目建设，监督实施中星化工公司搬迁，实现污染物集中控制和深度处理。实施农村环境连片整治项目，不断改善农村生活环境。加大汽车尾气、煤烟、扬尘等综合治理，确保空气环境质量优良率保持在85%以上，切实提高城市环境质量。通过坚持不懈的努力，确保人民群众生活在水清、天蓝、地干净的宜居环境之中！

（六）致力于保障和改善民生，全面发展社会事业。把解决民生问题摆在更加突出的位置，促进社会事业更快进步和公共服务进一步提升。今年政府将继续整合资金30多亿元再办一批民生工程，全心全意为人民群众办实事、谋福祉！

坚持优先发展教育。深入开展"教育均衡发展年"活动，推动教育改革试点，着力推进各级各类教育协调发展，加快义务教育均衡发展步伐和教育现代化建设进程，提升职业教育办学水平，实施学前教育行动计划，关心支持特殊教育，发展成人教育，完善涵盖终身的教育体系，使教育质量大幅提升。启动实施第二轮教育布局调整和校安工程，重点抓好五中、十四中、聋哑学校、张家湾小学迁建和四中扩建工程，推进三县示范高中建设，加快规划和实施一批城乡幼儿园项目，不断满足各个层次的教育需求。

提升公共卫生服务。巩固深化医药卫生体制改革及公立医院改革试点成果，完善城乡医疗服务体系，抓好海湖新区医院、市级综合（专科）医院及社区卫生服务中心（站）、乡镇卫生院等项目建设。进一步完善疾病预防控制体系，提高应对公共卫生事件的处置能力。加大医护人员培训和专业人才引进力度，不断提升医技水平，满足城乡群众就医需求。

推动创业带动就业。重点解决大中专毕业生、复转退伍军人就业及下岗失业人员再就业，全力做好创业及就业工作，构建和谐劳动关系，服务经济社会发展。全面启动综合性创业孵化基地建设，为创业者提供全方位综合性服务和优惠政策等扶持。积极鼓励、支持各类经济实体吸纳大中专毕业生就业，大力开展全民创业意识培养和创业能力培训活动，培训创业人员1万人，实现城镇失业人员再就业1.4万人。

提高社会保障水平。进一步提高城乡各类保障标准和补贴标准，不断满足不同层次群众的保障需求。关注低保边缘人群生活状况，建立低保对象保障与物价上涨补贴动态管理长效机制，提升低保对象生活水平。推行低保对象医疗救助"一站式"服务，逐步提高孤儿和无人抚养未成年人生活补助标准。构建"政府主导、平台运作、社会参与、股权多元"的公共租赁房建设新模式，建设各类城镇保障性住房13758套，实施农村危房改造5000户，奖励性住房17000户，不断改善城乡居民的住房条件。

继续加强物价调控。把稳物价保民生的政策一以贯之，抓好每个季度，抓住每个月份，使影响全市价格总水平高位运行的主要因素得到有效抑制。加强同省外蔬菜、生猪产区的联系，建立互利合作的产销关系，建成和发挥好300个社区直销菜店的作用，减少流通环节，降低流通成本。对人民生活影响较大的重点商品继续采取政府补贴、以奖代补、批零差率控制和最高限价，严格实行"四包四定"制度和市区联动机制，加大市场巡查力度，严厉打击价格违法行为，进一步规范市场经营秩序，确保全年价格指数继续回落。

创新社会管理机制。研究制定政府向社会组织转移公共服务职能和购买服务的优惠政策，鼓励和引导社会组织承接政府部分服务职能，参与社会管理。依托社区基础平台，建立组团式服务管理队伍，实行"定格、定人、定责、定流程、定奖惩"的网格化、精细化服务，实现公共资源共享化，业务流程规范化，社工服务高效化。全面启动市区居住小区物业管理标准化建设，使物业管理提升成为创新社会管理、提升社会服务的重要抓手。强化社会管理综合治理，推进"平安西宁"建设，着力完善社会治安防控、社会稳定风险评估、突发事件应急处置、矛盾纠纷排查化解、群众利益诉求表达和社会管理支撑保障六大机制。进一步完善城市公共安全体系建设，抓好重点行业和重点地区安全生产管理，加强食品药品监管，确保人民群众生产生活安全。完善各级救灾应急指挥系统，全面提升减灾救灾工作水平。做好"双拥"模范城市和民族团结进步示范区创建工作，进一步强化新时期宗教工作，不断巩固和发展平等、团结、互助、和谐的社会主义民族关系，确保社会和谐稳定。优先发展社会养老服务事业，制定和落实养老服务优惠政策，加快养老服务设施建设。继续加强人口计生工作，充分发挥惠民政策的引领激励作用，重点加强对流动人口的服务管理，努力稳定人口增长水平，不断提高人口素质。

（七）致力于文化大发展大繁荣，全面推进文化西宁建设。认真贯彻十七届六中全会精神，按照"因地制宜、发展平衡、网络健全、运行有效、惠及全民、量力而行"的原则，构建结构合理、门类齐全、科技含量高、富有创意、竞争力强的特色文化产业体系，不断满足广大人民群众的精神文化需求。

推动文化事业更加繁荣。加快制定文化事业发展规划，探索"市场化运作、企业化管理、产业化发展"的新模式，不断促进文化事业大繁荣，把西宁建成地域特色鲜明、经济效益良好、创新能力凸显、产业体系较为完善、文化整体实力和核心竞争力明显增强的青藏高原文化中心。以国家公共文化服务体系示范城市为载体，深入推进歌舞团、戏剧团体制改革，成立全市范围的群众文化协会，加快广播、电视制播分离进度，全面开展文体旅游活动，积极打造西宁文化品牌。注重非物质文化遗产的保护和传承，进一步开发老爷山花儿会、湟中"八瓣莲花"、湟源排灯等资源，彰显西宁历史文化魅力。

构建公共文化服务体系。坚持资源共享、综合利用，科学编制全市城乡公共文化设施建设规划，推进市文化艺术中心和区县文化馆、图书馆、乡镇综合文化站、村（社区）文化活动室建设。深入实施广播电视村村通、文化信息资源共享、农家书屋等文化惠民工程，扎实开展全民阅读、全民健身活动，推动文化科技卫生"三下乡"、科教文体法律卫生"四进社区"，建立以城带乡联动机制，不断丰富城乡人民群众精神文化生活。

着力发展特色文化产业。弘扬西宁文化特色，加快产业基地建设，精心组织实施一批文化产业重大工程。加快文化、体育、旅游产业融合发展，抓好文化产业园区、影视剧院、体育游乐场等项目建设。培育3——4个文化产业龙头企业，创作一批文化精品剧（节）目和全国知名的文化品牌。发展壮大广播电视、电影院线、出版发行、演艺、会展等传统文化产业，加快发展文化创意、数字出版、移动多媒体等新兴文化产业。加大财政对各类商业演出和公共文化演出的扶持力度，鼓励支持民营企业和民营资本进入文化产业，促进文化产业大发展。

扎实推进文明城市创建。围绕城市公共文明指数达到西部城市前列、实现"全国文明城市"创建任务的目标，以建设廉洁高效的政务环境、公平公正的法治环境、规范守信的市场环境、舒适便利的生活环境、安全稳定的社会环境、可持续发展的生态环境、健康向上的人文环境为重点，全面加强社会公德、家庭美德、职业道德、个人品德教育，大力弘扬西宁精神，提高市民的生活水平、文化素质和道德水准，提升城市的吸引力、凝聚力和综合竞争力，切实用政府工作的辛苦指数提高人民群众的幸福指数！

（八）致力于激发城市发展活力，继续扩大改革开放。积极探索重要领域和关键环节的改革，建立有利于科学发展的新机制。在更深的层次和更广的领域，全面推进改革开放，不断增强发展的活力和动力。

深化重点领域改革。进一步深化行政体制改革，健全科学、民主、依法决策机制，增强公共政策制定透明度和公众参与度。按照政事分开、事企分开和管办分离的原则，加快事业单位改革，推进文化卫生、城市公交等体制改革，让人民群众得到更多实惠。积极推进"政府雇员"制度，培养、引进和留住各类优秀人才。抓好融资体制改革，运用信托、债券等融资方式，创新城市建设项目筹融资运作模式，形成可持续的城市建设资金支持体系。

大力培育市场主体。集中力量，采取补贴和直接投入等措施，在园林旅游、文化体育、便民服务、设施农业等行业培育更多市场主体，重点扶持以市民创业为主导的微小企业，重点扶持市民开办家庭旅馆、餐厅等文化旅游服务业态，重点扶持公共服务市场化、社会化的有益探索，既缓解旅游压力，提升公共服务水平，更带动民众创业，促进和扩大就业。

提升对外开放水平。坚持"走出去"与"引进来"有机结合，继续拓宽利用外资的领域和渠道，注重提高利用外资的水平，更多地引进先进技术和管理经验。办好"夏都国际论坛"，加大对外向型企业的涉外服务力度。积极推进与发达地区乃至国际间的经济合作，加快构建内外联动、互利共赢、安全高效的开放型经济体系，让西宁的发展更好地融入国际国内的发展之中。

三、加强政府自身建设

全面完成2012年的各项工作任务，责任在肩，使命光荣。我们将牢固树立科学谋划、执政为民、加快发展、勤政廉政的意识，进一步加强政府自身建设，不断提高执政水平，真正做到为人民服务，对人民负责，受人民监督，让人民满意。

（一）注重调研，广聚民智，确保科学决策。坚持以科学发展观总揽全局，进一步解放思想，创新思路，紧紧抓住难得的发展机遇，克服多种不利因素，实现科学发展。深入开展调查研究，听民意，集民智，大力弘扬求真务实的工作作风，做广大群众"看得见"的干部，强化责任追究机制，不断完善重大事项听证、专家咨询、责任追究和重大建设项目工作例会制度，使各项决策更具科学性和前瞻性。

（二）接受监督，完善制度，确保依法行政。坚持民主集中制原则，主动接受市人大的法律监督和市政协的民主监督，依法履行政府职责，确保政府施政行为的法制化、民主化和科学化。及时公开群众关心和关注的事项及信息，接受群众和舆论的监督，不断提高政府公信力。

（三）善于创新，狠抓落实，确保高效执行。坚持从实际出发，以群众满意为标准，做出政绩、干出成效。坚持一线工作法，敢于直面矛盾，一个一个解决问题，一项一项落实工作。关注民生问题，特别是选准选好民生实事，尽快明确、及早开工，言必行，行必果！坚持用大开放的思路研究新形势，以大创业的动力解决新问题，以大发展的成果破解新瓶颈，以大和谐的局面推动全社会的创新！

（四）以身作则，廉洁奉公，确保勤政廉政。始终牢记"两个务必"，大力发扬艰苦奋斗，勤俭节约的优良作风。进一步健全惩治和预防腐败体系，强化对重点领域和重要岗位的监督，坚决制止和纠正损害人民群众利益的不正之风，努力打造一支人民满意的公务员队伍。

各位代表，抓住机遇，迎接挑战，创造西宁美好未来，是历史赋予我们光荣而神圣的使命。我们要在省委、省政府和市委的坚强领导下，进一步创新思路，科学谋划，艰苦奋斗，狠抓落实，加快实现"十二五"经济社会发展目标，为打造人民满意的生活之城、幸福之城，把西宁建设成更加繁荣、更加美丽、更加宜居的青藏高原现代化中心城市而努力奋斗！
